# SU-101 for the removal of pharmaceutical active compounds by the combination of adsorption/photocatalytic processes

**DOI:** 10.1038/s41598-024-58014-w

**Published:** 2024-04-03

**Authors:** Antonio J. Chacón-García, Sara Rojas, Erik Svensson Grape, Fabrice Salles, Tom Willhammar, A. Ken Inge, Yolanda Pérez, Patricia Horcajada

**Affiliations:** 1 Advanced Porous Materials Unit (APMU), IMDEA Energy Institute, 28935 Móstoles, Madrid Spain; 2 Department of Inorganic Chemistry, University of Granada, 18071 Granada, Spain; 3 Department of Chemistry and Biochemistry, Material Science Institute, University of Oregon, Eugene, OR 97403 USA; 4 Department of Chemistry – Ångström Laboratory, Uppsala University, 75120 Uppsala, Sweden; 5 ICGM, CNRS Université Montpellier, Montpellier, France; 6 Department of Materials and Environmental Chemistry, Stockholm University, 106 91 Stockholm, Sweden; 7 COMET-NANO Group, ESCET, Universidad Rey Juan Carlos, 28933 Móstoles, Madrid Spain

**Keywords:** SU-101, Photoactive Bi-MOF, Pharmaceutical active compounds, Water remediation, Combined elimination of several contaminants, Toxicological evaluation, Environmental sciences, Chemistry

## Abstract

Pharmaceutical active compounds (PhACs) are some of the most recalcitrant water pollutants causing undesired environmental and human effects. In absence of adapted decontamination technologies, there is an urgent need to develop efficient and sustainable alternatives for water remediation. Metal–organic frameworks (MOFs) have recently emerged as promising candidates for adsorbing contaminants as well as providing photoactive sites, as they possess exceptional porosity and chemical versatility. To date, the reported studies using MOFs in water remediation have been mainly focused on the removal of a single type of PhACs and rarely on the combined elimination of PhACs mixtures. Herein, the eco-friendly bismuth-based MOF, SU-101, has been originally proposed as an efficient adsorbent-photocatalyst for the elimination of a mixture of three challenging persistent PhACs, frequently detected in wastewater and surface water in ng L^−1^ to mg·L^−1^ concentrations: the antibiotic sulfamethazine (SMT), the anti-inflammatory diclofenac (DCF), and the antihypertensive atenolol (At). Adsorption experiments of the mixture revealed that SU-101 exhibited a great adsorption capacity towards At, resulting in an almost complete removal (94.1 ± 0.8% for combined adsorption) in only 5 h. Also, SU-101 demonstrated a remarkable photocatalytic activity under visible light to simultaneously degrade DCF and SMT (99.6 ± 0.4% and 89.2 ± 1.4%, respectively). In addition, MOF-contaminant interactions, the photocatalytic mechanism and degradation pathways were investigated, also assessing the toxicity of the resulting degradation products. Even further, recycling and regeneration studies were performed, demonstrating its efficient reuse for 4 consecutive cycles without further treatment, and its subsequent successful regeneration by simply washing the material with a NaCl solution.

## Introduction

Safe water, representing only 2.5% of the total water on Earth, is a limited resource vital for life. The increasing world population, along with rapid urbanization and increasing industrial activities have triggered the appearance of emerging organic contaminants (EOCs), now detected in most wastewater effluents at significant concentrations (ng·L^−1^–mg·L^−1^)^1,2^. In particular, the presence of the so-called pharmaceutical active compounds (PhACs) in wastewaters^3,4^ (e.g. antibiotics, endocrine disruptors, β-blockers, analgesic, non-steroidal anti-inflammatory drugs) is a serious global environmental concern^5^. As representative examples of persistent water pollutants: (i) the widely used veterinary antibiotics, sulfonamides (e.g. sulfamethazine (SMT)), have been detected in soil and water media, reaching a release of nearby 20,000 tonnes every year into the environment^6^ and being associated with the current antibiotic resistance crisis^7^; (ii) the extensively prescribed anti-inflammatory drug, diclofenac (DCF), has been recognized to cause decline and toxic effects not only in aquatic organisms^8,9^, but also in predators from secondary poisoning due to the consumption of food contaminated by DCF in surface waters from wastewater treatment plants (WWTPs)^10^; and (iii) atenolol (At), a β-blocker mediator used to treat high blood pressure and heart-associated chest pain, has been found in very high concentration in both the influents (295,700 and 33,106 ng·L^−1^ in India and Europe, respectively) and the effluents of WWTPs (519 and 7602 ng·L^−1^, respectively)^11^, negatively impacting on the aquatic environment^12^.

With this in mind, we propose the merging of an effective traditional method (i.e. adsorption) with an advanced oxidation process (i.e.*.* photocatalysis) to more effectively tackle water pollution. The combination of adsorption and photocatalysis is beneficial due to its simplicity, cost-effectiveness, eco-friendliness, and easy operation (i.e. batch or continuous flow). Many adsorbents such as biochar^13^, activated carbons^14^, zeolites^15^ or clays^16^ have been employed for the removal of EOCs. However, the use of adsorbents can be limited owing to (i) their preferential adsorption for only a type of contaminant, (ii) their limited adsorption capacity and (iii) their incomplete regeneration after saturation. Regarding photocatalytic processes, although conventional photocatalysts (e.g. TiO_2_, ZnO) have been popularly used in the removal of a single pollutant under UV–vis irradiation, some limitations (poor accessibility of adsorbate, high electron–hole recombination and large bandgaps) necessitates their modification for a visible-light response (i.e. doping^17^, co-doping^18^ or heterojunctions formation^19^). In this sense, metal–organic frameworks (MOFs) are versatile porous hybrid crystalline materials with a superior porosity and binding sites, which make them promising adsorbents and/or catalysts^20,21^. Liu et al. reported the selective uptake of DCF using the Cu-based MOF [Cu(BTTA)]_n_·2DMF (H_2_BTTA = 1,4-bis(triazol-1-yl)terephthalic acid), attaining a greater adsorption capacity than that achieved for graphene oxide, activated carbon or modified zeolites^22^. Also, some MOFs have exhibited photochemical properties adapted for water remediation, such as Ti-, Zr-, or Bi-based MOFs^23–25^ or their derivatives (e.g. post-synthesis modification, incorporation of metal nanoparticles), which are applied in an effort to enhance the visible-light photocatalytic activity and mitigate electron–hole recombination in the parent MOFs^26^.

Despite the growing interest of MOFs in water remediation by adsorption or degradation, it is only recently when we have originally proposed the combination of both adsorption and photocatalysis of several pollutants within a single highly porous and robust photoactive Zr-MOF^27^. This highlights its potential as both an effective adsorbent and an efficient photocatalyst. Other key aspects that remain still scarcely explored are the MOF stability under working conditions, the co-elimination of pollutants^28,29^ and the identification/toxicological evaluation of the degradation products^30^.

Within this context, the concomitant (adsorption/photocatalytic) removal of three challenging PhACs (i.e. SMT, DCF and At) by a single MOF is investigated here under relevant conditions, identifying and evaluating the degradation products. Thus, we propose for the first time the eco-friendly microporous bismuth(III) ellagate SU-101 (surface area > 400 m^2^·g^−1^ and pore size ~ 6.8 Å)^31^, with potential adsorption (i.e. high degree of porosity) and photoactive sites (i.e. a high-valent metal as Zr^4+^)^32^ and an outstanding thermal and chemical stability, as an efficient decontamination agent in water remediation. SU-101 has recently been used in promising applications: (i) adsorbing gases such as SO_2_ and H_2_S^31^ or CO_2_ from C_2_H_2_ and C_2_H_4_^33^ (ii) selectively adsorbing of Ni^2+^ over Co^2+^ ions^34^, (iii) catalysing the ring-opening alcoholysis of cyclohexene oxide^35^, or the CO_2_ cycloaddition reaction^36^ and (v) serving as a light-sensitive drug carrier^37^. Importantly, the recycling and regeneration of SU-101 upon water remediation tests is also studied.

## Experimental section

### Chemicals

All reagents were purchased and used without further purification. Ellagic acid (EA) reagent-grade (ACROS Organics, 97%), bismuth (III) acetate (Alfa Aesar, 99%), and glacial acetic acid (Merck, 100%) were used for the synthesis of SU-101. PhACs, used for the adsorption and photodegradation experiments, include atenolol (At; Sigma-Aldrich, ≥ 98%), sulfamethazine (SMT; Sigma-Aldrich, ≥ 99%) and sodium diclofenac (DCF; Sigma-Aldrich, ≥ 98). Formic acid (FA; Thermo Scientific, ≥ 98%) and acetonitrile (ACN; J.T. Baker, HPLC-grade) were used for the preparation of HPLC mobile phases. For the preparation of the phosphate buffered saline (PBS) solution, sodium anhydrous phosphate dibasic (ACROS Organics, 98%), sodium phosphate monobasic anhydrous (ACROS Organics, 98%), orthophosphoric acid (ACROS Organics, ≥ 85%) and Milli-Q water were used. Isopropyl alcohol (VWR, 99%), p-benzoquinone (ACROS, ≥ 98%), disodium ethylenediaminetetraacetate dihydrate (Na_2_EDTA, Fisher Scientific, 99.5%) and silver nitrate (ACROS Organics, 99.9%) were employed for active species trapping tests.

### Synthesis of SU-101

SU-101 was synthesised following the procedure reported by Svensson-Grape et al.^31^.

### General instrumentation

Powder X-ray diffraction patterns (PXRD) were acquired using an Empyrean (PANALYTICAL) diffractometer from 3 to 35° (2Θ), with a radiation source of CuKα (Niβ-filter, λ = 1.5406 Å) equipped with a PIXce3D detector and operating at 45 kV and 40 mA. Fourier transform infrared (FTIR) spectra were collected from 1000 to 4000 cm^−1^ in a Thermo Scientific Nicolet 6700 spectrophotometer, employing the attenuated total reflectance (ATR) mode with 64 accumulations per scan. The UV–visible spectrum was obtained using a PerkinElmer Lambda 1050 UV–visible-NIR spectrometer equipped with an integrating sphere, operating in diffuse reflectance mode with BaSO_4_ as the reference material. The bandgap (E_g_) was estimated from a Tauc plot^38^.

### HPLC analysis

The identification and quantification of At, SMT and DCF, as well as, the MOF constitutive ligand, EA, were performed using a high-performance liquid chromatography (HPLC) Jasco LC-4000 series system, equipped with a photodiode array detector (PDA) MD-4015. Isocratic and isothermal (298 K) conditions were employed for the analysis of all the samples in a purple ODS reverse-phase C18 column (5 µm, 4.6 × 150 mm), using a flow rate of 1 mL·min^−1^ and a volume injection of 30 µL.

The remaining At concentration was determined using a mobile phase based on a mixture of 90:10 PBS:ACN, at a retention time (rt) of 4.6 min and an absorption maximum at 227 nm (Fig. S1). For the preparation of a 0.04 M PBS buffer solution (pH = 2.5), 0.02 mol (2.4 g) of NaH_2_PO_4_ and 0.02 mol (2.84 g) of Na_2_HPO_4_ were dissolved in 1 L of Milli-Q water, and then, the pH was adjusted with H_3_PO_4_ (≥ 85% v/v). For the DCF analysis, the mobile phase was composed of a mixture of 70:30 ACN:FA (10% v/v), identifying the peak at a rt of 3.6 min and an absorption maximum at 275 nm (Fig. S2). For the SMT determination, the mobile phase was based on a mixture of 35:65 ACN:H_2_O, with a rt of 3.0 min and at 263 nm (Fig. S3). The released EA linker (Limit of Detection LOD = 0.06 ppm) was analysed using a mobile phase with a composition of 70:20:10 H_2_O:ACN:FA (10% v/v), with a rt of 5.4 min and an absorption maximum at 280 nm (Fig. S4).

### LC–MS analysis

The degradation mechanisms of DCF, SMT, and At were determined by HPLC–MS in an Agilent 1260 infinity II (Agilent Technologies, Santa Clara, CA, USA), equipped with a single quadrupole mass spectrometer (MS) detector InfinityLab LC/MSD-iQ (Agilent Technologies, Santa Clara, CA, USA) coupled to an API-Electrospray Source, working in positive and negative ion mode and analysing samples in a mass range of 10–400 m/z for SMT and 40–400 m/z for DCF and At. The identification of the degradation products of DCF and SMT was carried out with a mobile phase consisting of a mixture of a solution of 10% FA ACN: H_2_O (35:65) and in the case of At using a linear gradient (0–5 min), from a solution of 10% FA H_2_O:MeOH (95:5) to a solution of 10% FA H_2_O:MeOH (5:95). In both cases, the mobile phase was delivered at a flow rate of 0.5 mL·min^−1^ at 35 °C and using an InfinityLab Poroshell 120 EC-C18 column (2.1 × 150 mm; 2.7 Micron; Agilent Technologies, Santa Clara, CA, USA)**.**

### Lab-scale adsorption and photodegradation experiments of a single and a mixture of contaminants in tap water

Tap water solutions of the selected PhACs (At, DCF, and SMT) were prepared separately, and in mixtures at spiked concentrations of 10 mg·L^−1^, to evaluate the adsorption and photocatalytic properties of SU-101. Using a 10 mL glass reactor, 4 mg of SU-101 were placed in contact with 4 mL of a contaminated solution (containing a single contaminant or a mixture of At, DCF, and SMT) under continuous stirring. The suspension was maintained under darkness for adsorption experiments or exposed to visible light irradiation for photocatalytic tests. A 300 W Xe lamp (Oriel instruments OPS-A500), equipped with a cut-off filter at 420 nm, was used as visible light source. Aliquots of 100 µL were taken at intervals of 0, 0.25, 0.5, 1, 2, 4, 5, and 24 h and analysed by HPLC. All experiments were done in triplicates and the standard deviation was calculated. Additionally, the linker release was monitored by HPLC in order to study the stability of SU-101 under working conditions.

### Regeneration of SU-101

The regeneration of At-loaded SU-101 was realized using a saturated solution of NaCl. Firstly, in an attempt to saturate the SU-101 with At, 4 mg of SU-101 were suspended in 4 mL of a highly concentrated solution of At (1000 mg·L^−1^) under stirring and dark for 4 h. Then, the At-loaded material was filtered under vacuum, and the supernatant liquid evaluated by HPLC in order to determine the remaining concentration of At. For the regeneration process, the At-loaded SU-101 was dispersed in 4 mL of a NaCl-saturated solution under continuous stirring and the At release was quantified at time intervals of 5, 10, 15, and 30 min.

### Recyclability of SU-101

The reusability of SU-101 was tested using a mixture of the three selected PhACs (At, DCF and SMT) in tap water and preserving the experimental conditions previously used. After each cycle of 4 h, the solid was separated by centrifugation and, without further treatment, the material was dispersed in a fresh pollutant mixture (At, DCF and SMT) in tap water. Finally, the resulting liquids were analysed by HPLC to determine the remaining concentration of each PhAC. In addition, the structural stability of SU-101 was studied after each cycle by PXRD, as well as the linker release was monitored using HPLC.

### QSAR calculations

Quantitative structure activity relationship (QSAR) studies concerning acute toxicity (oral rat LD_50_), bioconcentration factor, developmental toxicity, and mutagenicity were performed by the Toxicity Estimation Software Tool (T.E.S.T) developed by the U.S. Environmental Protection Agency (EPA).

### Monte Carlo calculations

In order to determine the plausible distribution of the confined molecules in the SU-101 structure, Monte Carlo simulations were performed using Materials Studio^39^. Considering the crystal structure obtained experimentally^31^, partial charges were calculated after a geometry optimization by DMol^3^ (available in Materials Studio) following the Mulliken scheme for both the solid and the contaminants. For the convergence, energy convergence, gradient convergence and displacement convergence were fixed at 10^–5^ Ha, 2·10^–3^ Ha/Å and 5·10^–3^ Å, respectively. The so-obtained partial charges distribution allows us to determine the electrostatic part of the energy for Monte Carlo simulations. The van der Waals interactions were then evaluated using the Lennard–Jones parameters issued from Universal Force Field (UFF)^40^. The UFF parameters were combined following the Lorentz–Berthelot rules during Monte Carlo simulations. The electrostatic interactions were computed using the Ewald summation, while the short-range contributions were determined using a cut-off for Lennard–Jones parameters fixed at 12.5 Å. For Monte Carlo calculations, we imposed a pressure of contaminants equal to 10^4^ kPa, sufficient enough to reach the saturation of the loading, considering a SU-101 multi-cell structure (corresponding to 2 × 2 × 5 structure based on the experimental unit cell) with a constant unit cell volume and temperature (300 K). Monte Carlo calculations consisted of 20 million steps of equilibration and 3 million steps for production.

The results obtained from Monte Carlo simulations were presented as (i) the adsorption heat, which reproduces the affinity of the MOF by the investigated contaminants, and (ii) the distribution of the contaminant molecules in the pores, allowing us to determine the main interaction sites.

## Results and discussion

### Adsorption of PhACs

**Figure 1:**
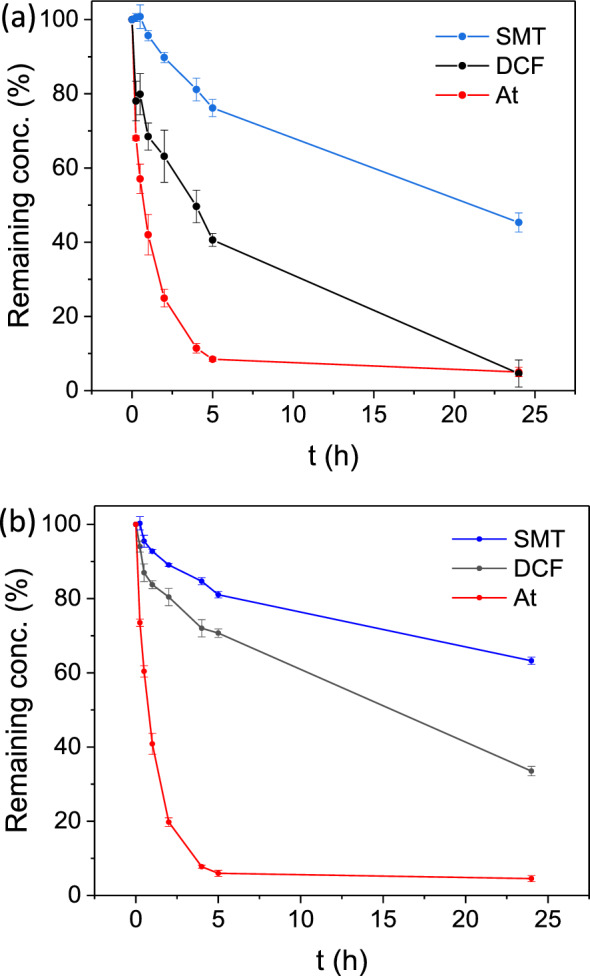
Adsorption of PhACs by SU-101 of (**a**) single PhACs and (**b**) a mixture of PhACs in tap water solutions. Sulfamethazine (SMT, in blue), diclofenac (DCF, in black), and atenolol (At, in red).

For comparative purposes and considering the found high concentration of these three contaminants in water environments (up to 8 mg·L^−1^ for At, and 1 mg·L^−1^ for SMT and DCF^41–44^) as well as their accurate detection, the initial concentration of each PhAC was fixed to 10 mg·L^−1^. Practically, SU-101 was put in contact with separated tap water solutions containing a single PhAC (SMT, At or DCF; 10 mg·L^−1^) and the adsorption of each contaminant was determined by HPLC (see experimental section). Remarkably, SU-101 was able to adsorb the three PhACs (see Fig. 1a), reaching a removal in only 5 h of 91.5 ± 0.6, 59.4 ± 1.7 and 23.8 ± 2.3% for At, DCF and SMT, respectively. Comparing our results with previous works (see Table S1), the At adsorption capacity of SU-101 is remarkably higher than that reported with carbon-based materials (e.g. activated carbon^45^, carbon nanotubes (MWCNTs)^46^) or magnetic powdery acrylic polymer (MPAP)^47^, and comparable with the use of modified MOFs (e.g. KOH@Ni8BDP6)^48^ or modified carbon nanotubes (e.g. M-MWCNTs)^46^.

The adsorption of the contaminants can be influenced by several physicochemical considerations, such as geometry and interactions. It should be mentioned that the investigated loadings, corresponding to the environmental conditions, and the resulting adsorption amounts are in the same order of magnitude as the saturation amounts estimated from Monte Carlo simulations (35, 40 and 45 mg g^−1^ for At, DCF and SMT, respectively). The higher adsorption of At on SU-101 could be partially explained by its more lineal structure compared to SMT and DCF molecules. The Monte Carlo calculations unambiguously show a preference of SU-101 for At (for which the adsorption heat is close to 200 kJ·mol^−1^, while the values obtained for DCF and SMT are closed to 160 kJ·mol^−1^). This comparison allows to conclude that SU-101 has a stronger affinity for At compared to DCF and SMT. One should keep in mind, however, that the Monte Carlo simulations were performed without taking into account the effect of solvent molecules (which can modify the balance of MOF-contaminant interaction). Further, the secondary amine of the At (pKa = 9.4), which would be protonated at the tap water pH (~ 6), and the slightly negative surface of SU-101 (ζ-potential = − 10 mV; probably associated to the presence hydroxyl groups on its surface^31^), could promote the formation of specific interactions (e.g. electrostatic, hydrogen bonds). In contrast, DCF would be in its anionic form in tap water (pKa = 4.0 carboxylic group)^49^. Therefore, the formation of electrostatic interactions should not be favoured, except for hydrogen bonds between the –Cl or –COO^−^ groups from DCF and the –OH or H_2_O from SU-101. In the case of SMT, its neutral charge (amine group, pK_a1_ = 2.6) and its highly distorted geometry might hinder its adsorption inside the MOF pores. SMT could interact by its amine group with the carbonyl groups of the ellagate ligand of SU-101 or the –SO_2_ group with the –OH from SU-101. And finally, in all cases, pi-stacking effect can occur between contaminant molecules and SU-101 framework. All the considered interactions are depicted in Fig. S5.

To assess the SU-101 stability, samples after the adsorption experiments were analysed by PXRD and FTIR, revealing that the crystallinity was not affected by the presence of the PhACs or the inorganic species present in tap water (see Figs. S6 and S7). Additionally, the leaching of the SU-101 constitutive linker (EA) was monitored by HPLC, revealing no detectable release to the media (limit of detection-LOD > [EA]) and thereby confirming the high hydrolytic stability of SU-101.

In a second step, considering that pollutants are usually found in wastewater as complex mixtures with other organic and inorganic compounds, the adsorption capacity of SU-101 was also studied using a mixture of the three contaminants (At, DCF, and SMT) in tap water. As can be seen in Fig. 1b, the combined adsorption is in accordance with respect to what was observed in the individual adsorption experiments. Thus, SU-101 shows a preferential adsorption of At (94.1 ± 0.8% in 5 h), which hampers the DCF and SMT retention (29.3 ± 1.1 and 18.9 ± 0.9% for combined adsorption *vs*. 59.4 ± 1.7 and 23.8 ± 2.3% in individual adsorption), supporting the relevance of electrostatic interactions in the adsorption mechanism, as previously discussed. The At preference binding on anionic MOFs was previously observed by some of us^27^.

The regeneration of the adsorbent often becomes a challenging task, limiting its reusability in successive cycles and its practical use for large-scale process. In this sense, the efficiency of the desorption process mainly depends on the strength of the interaction between the adsorbent and the adsorbate. Regeneration mechanisms can be classified into thermal and non-thermal processes. The first involves high energy requirements such as temperature, vacuum, electrical induction, microwave regeneration, or, in some cases, the combination with solvent-assisted methods, being costly and energy-consuming. Conversely, non-thermal methods are a cost-efficient alternative, being the most common solvent or solution-based regeneration treatment^50^. In this work, the regeneration of At-loaded SU-101 was assessed by a fast, easy and eco-friendly process consisting of washing the material with a saturated sodium chloride solution. Firstly, SU-101 was loaded with At, reaching a loading of 0.09 ± 0.01 mg At per mg of SU-101. Then, the At-loaded material was dispersed in a NaCl-saturated solution at room temperature for the regeneration (see experimental section). The process resulted in a high At release (66.0 ± 2.0%) in only 5 min (Fig. S8a), proving an efficient way to regenerate the MOF. Besides, the PXRD pattern of the regenerated SU-101 reveals no significant structural changes (see Fig. S8b), supporting its potential regeneration.

### Photodegradation of PhACs

As a control, blank photodegradation experiments of PhACs were performed in absence of the MOF under light irradiation for a total of 24 h (see Fig. S9), confirming that there was no significant degradation of the contaminants. The photocatalytic activity of SU-101 was then evaluated for degrading the selected PhACs under both an individual and a combined process. The photodegradation experiments were conducted under comparable conditions to those for adsorption but under visible light irradiation. Note here that to simulate real treatment conditions, the experiments were carried out in tap water and without previously reaching the adsorption–desorption equilibrium.

**Figure 2:**
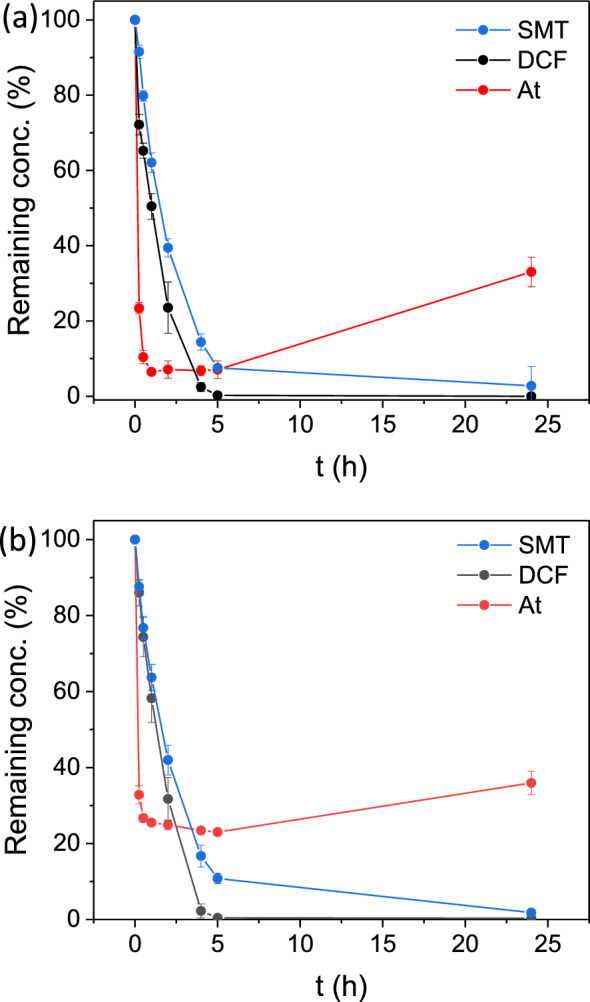
Photodegradation experiments with SU-101 using (**a**) individual PhAC solutions and (**b**) mixtures of the three PhACs. SMT (blue), DCF (black) and At (red).

The photodegradation results are shown in Fig. 2a, and reveal an outstanding removal efficiency of DCF and SMT (i.e. 99.6 ± 0.4% and 92.5 ± 0.5% in 5 h, respectively). Besides, DCF and SMT photodegradation can be fitted to a first order kinetic, with constants (k) of 1.04 and 0.51 h^−1^, respectively (see Fig. S10). SU-101 shows a superior visible-light photodegradation activity of SMT in comparison with benchmarked photocatalysts (e.g. TiO_2_^51^, g-C_3_N_4_^52^ or MIL-53(Fe)^53^; Table S2). Also, the DCF removal is more efficient than that of MOF composites (e.g. Fe_3_O_4_@MIL-100(Fe)^54^) and comparable with that of Ti-based photocatalysts such as TiO_2_/g–C_3_N_4_^55^ or NH_2_-MIL-125(Ti)^56^. Note here however that, in the latter case, the photodegradation was performed under UV–vis, making SU-101 (Eg = 2.67 eV; see Fig. S11) competitive as a simpler and very robust visible light catalyst (see Table S2). In the case of At, SU-101 also exhibited an exceptional removal efficiency (89.6 ± 1.8% in only 30 min). However, it is worth noting that the At elimination may be associated to a pure adsorption process since: (i) the maximum removal efficiency is similar to the one obtained under dark conditions, surpassing 90% of At removal in 5 h (see Fig. S12); and (ii) the concentration of At in the medium was increased at 24 h, presumably due to a partial At desorption following an increase of temperature from the prolonged visible light irradiation. It is important to note that At has been identified as a more persistent contaminant compared toother PhACs^57^, which is in line with the results of the photodegradation trials supporting its difficult degradation.

The simultaneous photocatalytic degradation of At, DCF, and SMT by SU-101 was also accomplished (Fig. 2b), reaching exceptional removals of DCF and SMT similar to those of single degradation experiments (99.6 ± 0.4% and 89.2 ± 1.4%, respectively). Photodegradation kinetics were fitted to a first order reaction and k-values of 1.11 and 0.43 h^-1^ for DCF and SMT, respectively, with no significant variations compared with individual experiments (see Fig. S10). In contrast, the At removal efficiency by SU-101 decreases in comparison with the single At removal (77.0 ± 0.4% vs. 93.0 ± 2.3% in 5 h). This could be explained by the competitive adsorption of the compounds generated from the DCF and SMT degradation, limiting the adsorption of At molecules.

### Reusability and stability of SU-101 under irradiation

**Figure 3:**
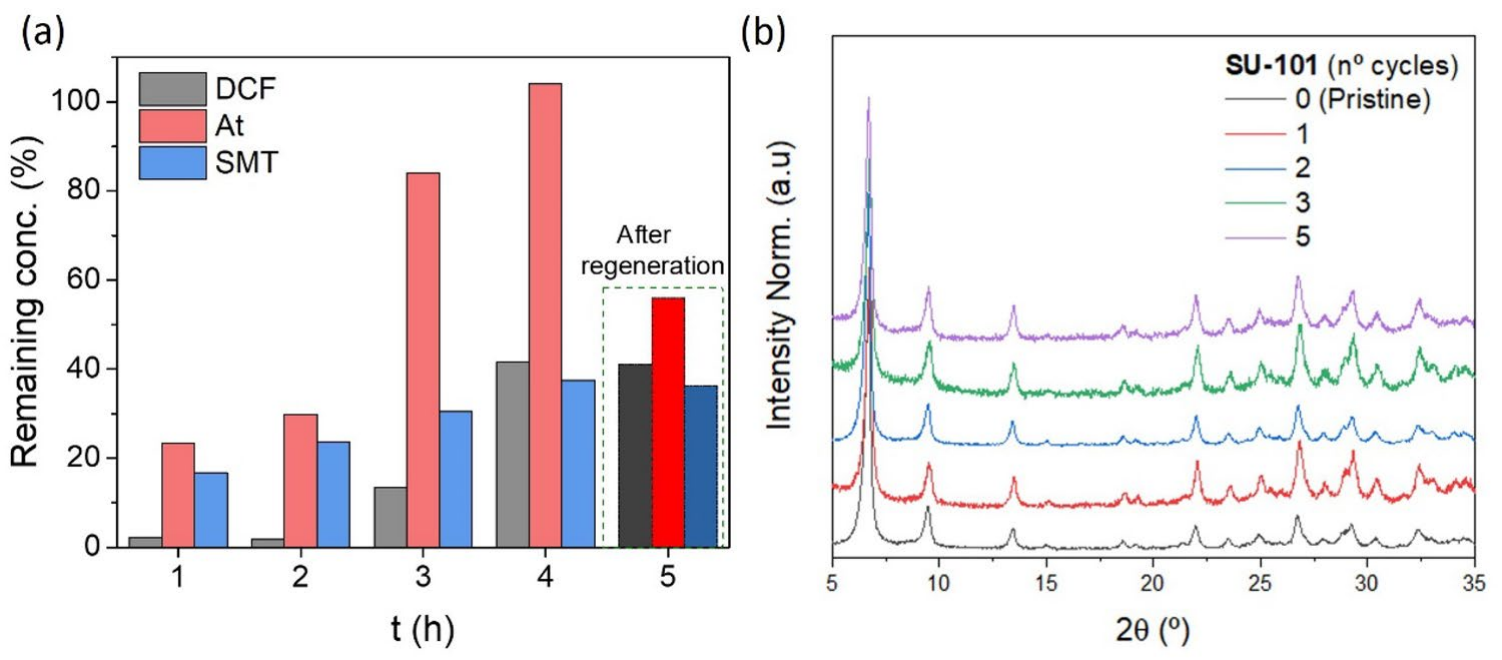
(**a**) Photodegradation cycles of a mixture of PhACs of 4 h each using SU-101 (DCF: grey, At: red and SMT: blue). (**b**) PXRD patterns of SU-101 after visible light irradiation upon different cycles. A regeneration step was performed after the 4th cycle.

The reusability of SU-101 was also investigated after the simultaneous photodegradation of the three selected PhACs without further treatment. The results revealed a good reusability and stability of SU-101, degrading more than 50% of DCF and SMT in 4 cycles of 4 h each (Fig. 3a). After these cycles, in an attempt to remove the adsorbed species (i.e. At, SMT, DCF, degradation products), the MOF was regenerated by simply washing with a NaCl-saturated solution and deionized water, allowing the reuse for an additional 5th cycle and recovering around the 50% of the initial At removal efficiency. Further, upon the regeneration and the 5th cycle, the structural and chemical integrity of SU-101 was confirmed by PXRD (Fig. 3b) and HPLC analysis (linker release).

### Photodegradation pathways and proposed mechanism

**Figure 4:**
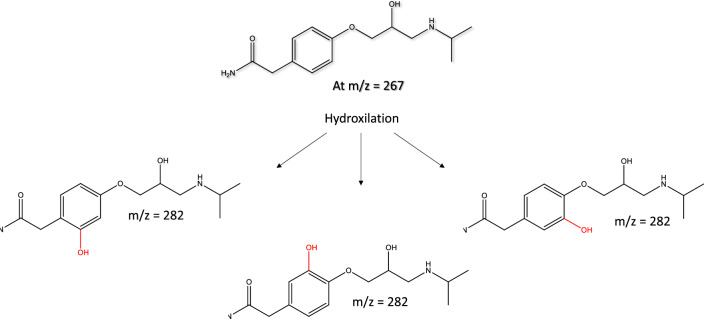
Potential degradation pathways of At.

**Figure 5:**
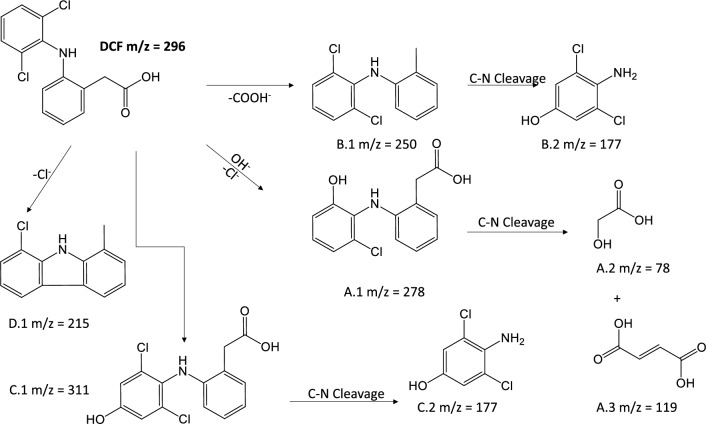
Proposed degradation pathways of DCF.

**Figure 6:**
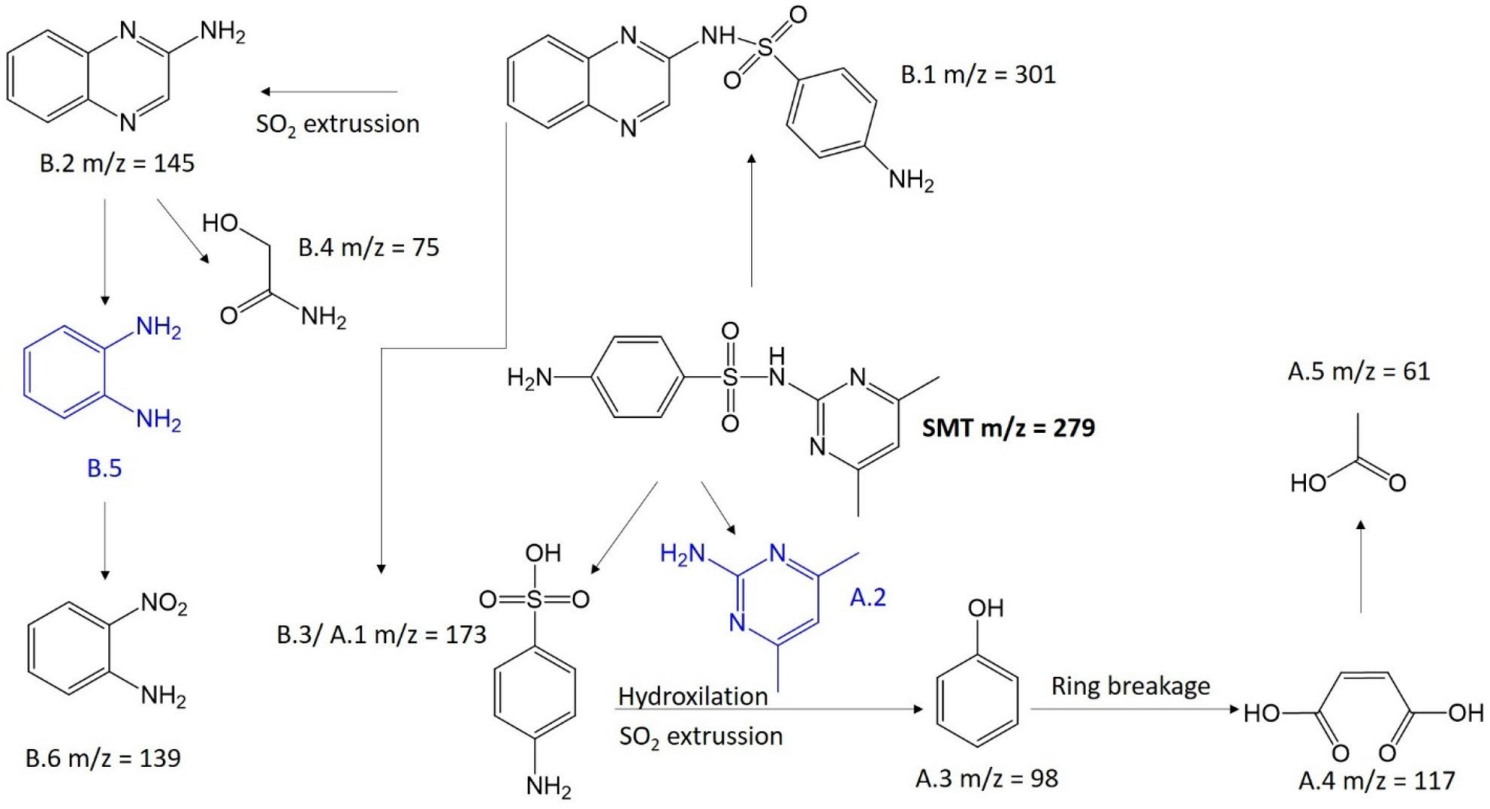
Proposed degradation pathways of SMT. Fragments in blue correspond with intermediaries which could be generated during the photocatalytic process.

To shed some light on the degradation pathways of DCF, SMT and At (Figs. 4, 5 and 6), their potential degradation products were identified using LC–MS analysis (Figs. S13, S14, S16 and S18). Even further, the potential toxicity of the degraded compounds was assessed by QSAR calculations with T.E.S.T software (Tables S3–S5).

*Atenolol*. The potential degradation pathways of At are shown in Fig. 4. Within 24 h, a LC MS peak related to At (m/z = 226) appears with a high relative intensity (Fig. S14), confirming that the removal of At is mainly due to a pure adsorption process. Additionally, the At molecule might suffer a slight transformation by the hydroxylation of the phenyl group, which can result in the formation of 2-[2-hydroxy-4-[2-hydroxy-3-(propan-2-ylamino)propoxy]phenyl]acetamide (m/z = 282), as previously reported^58,59^. The acute toxicity and bioconcentration of the transformed products, estimated by T.E.S.T., are lower than those calculated for At (Table S3 and Fig. S15).

*Diclofenac.* The degradation of DCF arises with the formation of the 2-[2-(2-chloro-6-hydroxyanilino)phenyl]acetic acid (A.1 m/z = 278) that results from the dechlorination and further hydroxylation of DCF (Fig. 5). Then, the process proceeds with the formation of glycolic acid (A.2 m/z = 78) and fumaric acid (A.3 m/z = 119). The presence of these species at the early stages might be related to the main photodegradation pathway.

As a secondary transformation, a lower proportion of DCF seems to be converted into the 2,6-dichloro-N-(2-methylphenyl)aniline (B.1 m/z = 250) by decarboxylation and subsequent C-N excision to give the 4-amino-3,5-dichlorophenol (B.2. m/z = 177) at 4 h. The formation of the fragment B.2 can also be ascribed to the C-N cleavage of the 2-[2-(2,6-dichloro-4-hydroxyanilino)phenyl]acetic acid (C.1 m/z = 311) at 2 h. Additionally, the presence of a low-intensity fragment at m/z = 215 can be due to the possible transformation of DCF into 1-chloro-8-methyl-9H-carbazole (D.1 m/z = 215)^60^. After 24 h, the main degradation compounds found in the solution correspond to the A.2 (higher proportion) and A.3 products (less proportion). The proposed degradation pathway is in good agreement with that one reported by Wei et al. where DCF was degraded by bio-Pd/Fe@Fe_3_O_4_ nanoparticles via the Fenton reaction^61^.

Interestingly, the toxicity estimation of A.2 and A.3 fragments revealed a lower acute toxicity in comparison with DCF (Table S4 and Fig. S17). Further, attending to their lower bioaccumulation factor, A.2 and A.3 fragments are considered as non-bioaccumulative and, in contrast to with DCF, both are non-mutagenic.

*Sulfamethazine.* Figure 6 summarizes the proposed degradation pathways of SMT in which the main way occurs by the scission of its S···N bond. The degradation continues through SO_2_ extrusion from sulfanilic acid (A.1) and subsequent hydroxylation to phenol (A.3, m/z = 98). Then, phenol A.3 was transformed into succinic acid (A.4, m/z = 117) by aromatic ring breakage and finally decomposed into acetic acid (A.5 m/z = 61). The presence of an intense peak corresponding with succinic acid (A.4) at 0.25 h supports a fast degradation of SMT. Besides, the proposed degradation pathways are in agreement with a previous work reporting the degradation of a similar SMT derivative (sulfadiazine) using the red mud powders and persulfate system^62^.

Although in a lower proportion, another secondary degradation pathway might be considered. Following pathway B, the (4-amino-N-(quinoxaline-2-yl) benzene sulphonamide (B.1 m/z = 301) is produced from SMT at 0.25 h^63^. The degradation may then continue through the conversion of B.1 into 2-amino quinoxaline. B.1 can be degraded into 2-hydroxiacetamide (B.4 m/z = 74) and 2-nitroaniline (B.6 m/z = 139), which can be produced by the fast oxidation of the intermediate o-phenylenediamine.

After 24 h, the resulting degradation compounds ordered according to the relative intensity of the peak are A.5 > A.4 > A.3 > A.1 > B.6. Interestingly, LC–MS analysis shows the formation of many low mass molecular compounds, indicating an efficient SMT photodegradation (see SI page S17). Notably, these degradation products are safer than the antibiotic SMT, as supported by the T.E.S.T. calculations (Table S5 and Fig. S19).

**Figure 7:**
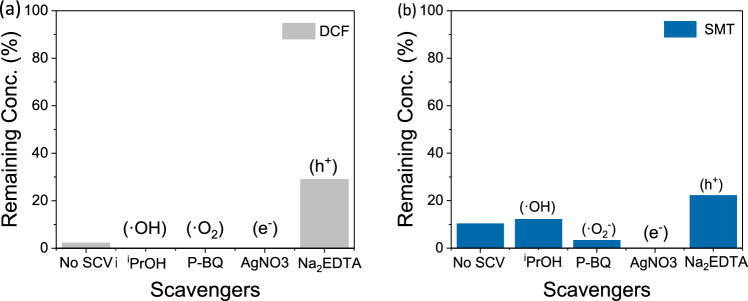
Photodegradation experiments of (**a**) DCF and (**b**) SMT in presence of scavengers.

As previously determined, the photodegradation of contaminants is accompanied by the formation of degradation products as a consequence of the fragmentation of the target molecules. Thus, reactive oxygen species (ROS; e.g. hydroxyl radical (^·^OH), superoxide anion radicals (^·^O_2_^−^)), and active species or precursors for ROS (e.g. positive holes (h^+^) and electrons (e^−^)) likely play a crucial role in the degradation. In an attempt to elucidate the photodegradation mechanism and the main reactive species involved in the photocatalytic process, different scavengers were employed during the photocatalytic reaction. The effect of scavengers such as AgNO_3_, p-benzoquinone, isopropanol, and Na_2_EDTA was investigated to quench e^−^, ^·^O_2_^−^, ^·^OH, and h^+^, respectively. Figure 7 shows the DCF and SMT photodegradation in the mixture of PhACs and in the presence of each scavenger (10 mM), suggesting that the holes (h^+^) are the primary species involved in the degradation process.

## Conclusions

To target the pressing environmental and health concern of PhACs in water, emergent technologies such as adsorption and/or photocatalysis are being developed. Among them, the robust eco-friendly SU-101 material is proven here as a promising alternative, through the combination of adsorption and photocatalysis, surpassing the capabilities of other previously tested decontamination agents (including TiO_2_, g-C_3_N_4_ or MIL-53(Fe) or Fe_3_O_4_@MIL-100(Fe)). Combining its high regular porosity with active sites, it was able to treat a mixture of three challenging PhACs in complex water (tap water). Thus, while the antihypertensive atenolol was mainly removed by adsorption, the antibiotic sulfamethazine and the anti-inflammatory diclofenac were concomitantly photodegraded under visible light into safer low-molecular weight products as evidenced by QSAR toxicity studies. Further, the outstanding chemical and structural stability of SU-101 allows its recyclability and regeneration without a significant loss of the simultaneous degradation of diclofenac and sulfamethazine. These results open a large avenue of possibilities to use SU-101 as a potent two-in-one adsorbent-photocatalyst not only in water remediation but also in other environment and energy-related applications, including air remediation, CO_2_ reduction or water splitting.

### Supplementary Information

Supplementary Information.

## Data Availability

Most of the data generated or analysed during this study are included in this published article [and its supplementary information files]. Some of the data used or analysed during the current study available from the corresponding author on reasonable request, but restrictions apply to the availability of these data, which were used under license for the current study, and so are not publicly available.
