# Supplementary material for: SU-101 for the removal of pharmaceutical active compounds by the combination of adsorption/photocatalytic processes
Source: Sci Rep. 2024 Apr 3;14:7882. doi: 10.1038/s41598-024-58014-w (PMC10991395; doi:10.1038/s41598-024-58014-w)
Supplement: Supplementary file 1 — Supplementary Information. [file 41598_2024_58014_MOESM1_ESM.docx]

**Supplementary information**

**SU-101 for the removal of pharmaceutical active compounds by the combination of adsorption/photocatalytic processes**

Antonio J. Chacón-García^1^, Sara Rojas^1,2^, Erik Svensson Grape ^3^, Fabrice Salles^4^, Tom Willhammar^3^, A. Ken Inge^3^, Yolanda Pérez^1,5,*^, Patricia Horcajada^1,*^

*^1^Advanced Porous Materials Unit (APMU), IMDEA Energy Institute, E-28935 Móstoles, Madrid, Spain;*

*^2^Department of Inorganic Chemistry, University of Granada, Granada, 18071, Spain;*

*^3^Department of Materials and Environmental Chemistry, Stockholm University, Stockholm 106 91, Sweden*

*^4^ ICGM, CNRS Université Montpellier, Montpellier, France*

*^5^Departamento de Biología y Geología, Física y Química Inorgánica, ESCET, Universidad Rey Juan Carlos, 28933 Móstoles, Madrid, Spain*

**Table of contents**

**Fig. S1, S2, S3, S4** HPLC identification, UV-Vis spectrum and standard calibration curve for At, DCF, SMT and EA **3-4**

**Table S1** Reported adsorbent materials for the removal of At**5**

**Fig. S5** Plausible organization of the contaminant molecules in SU-101 for At, DCF and SMT.

**Fig. S6** PXRD patterns of pristine SU-101 and SU-101 after individual PhAC adsorption experiments with At, SMT and DCF**7**

**Fig. S7** FT-IR spectra of SU-101 after individual PhAC adsorption experiments in comparison with the spectra of free DCF, SMT and At compounds**8**

**Fig. S8** At adsorbed and desorbed amount on SU-101 and PXRD patterns of SU-101 before and after NaCl treatment.**9**

**Fig. S9** Photodegradation of the selected PhACs in absence of SU-101 under visible light irradiation**10**

**Fig. S10** Kinetic linear fitting plots of the photodegradation studies using individual and mix solutions of PhACs**10**

**Fig. S11** Band-gap estimation of SU-101 considering an indirect allowed transition **11**

**Table S2** Photodegradation of DCF and SMT using other reported photocatalysts and the used experimental conditions**11**

**Fig. S12** Comparison between adsorption and photodegradation experiments using isolated samples of At**12**

**Fig. S13** Tap water MS used during the photodegradation experiments**12**

**Fig. S14** MS of At photodegradation experiments at different time intervals **13-16**

**Table S3** Estimated toxicity of At degradation products using the Toxicity Estimation Software Tool (T.E.S.T) based on QSAR model.**17**

**Fig. S15** Comparison of acute toxicity, bioconcentration factors, developmental toxicity and mutagenicity of At generated compounds**17**

**Fig. S16** MS of DCF photodegradation experiments at different time intervals **18-21**

**Table S4** Estimated toxicity of DCF degradation products using the Toxicity Estimation Software Tool (T.E.S.T) based on QSAR model.**22**

**Fig. S17** Comparison of acute toxicity, bioconcentration factors, developmental toxicity, and mutagenicity of DCF generated compounds**22**

**Fig. S18** MS of SMT photodegradation experiments at different time intervals **23-26**

**Table S5** Estimated toxicity of SMT by-products using the Toxicity Estimation Software Tool (T.E.S.T) based on QSAR model.**27**

**Fig. S19** Comparison of acute toxicity, bioconcentration factors, developmental toxicity, and mutagenicity of SMT generated compounds**27**

**References28**

***Atenolol (At)***


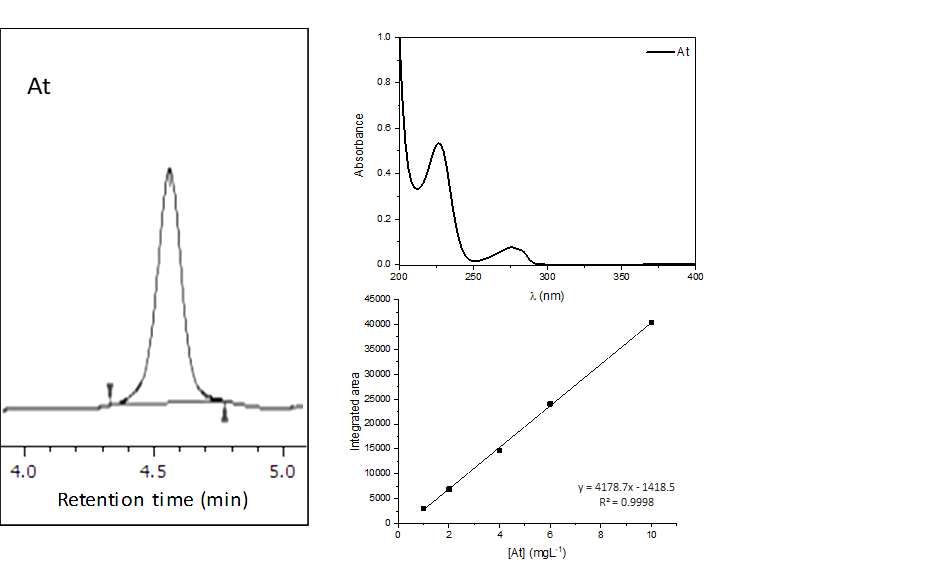


(c)

(b)

(a)

**Fig. S1.** (a) HPLC identification, (b) UV-Vis spectrum and (c) standard calibration curve for At.

***Diclofenac (DCF)***

(a)


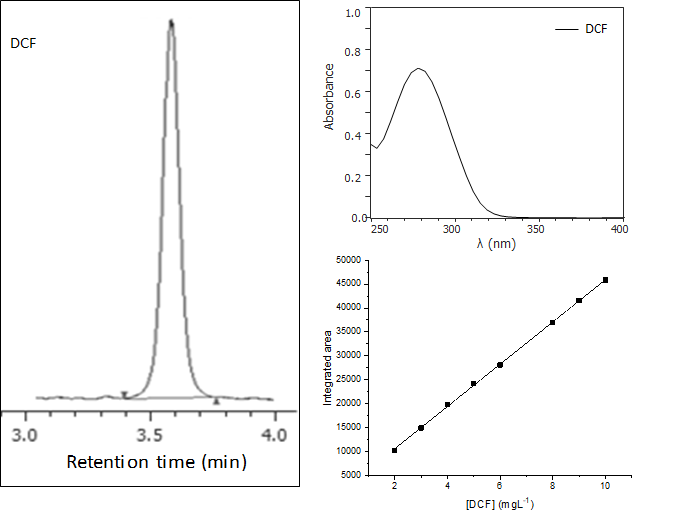


(c)

(b)

**Fig. S2.** (a) HPLC identification, (b) UV-Vis spectrum and (c) standard calibration plot for DCF

***Sulfamethazine (SMT)***


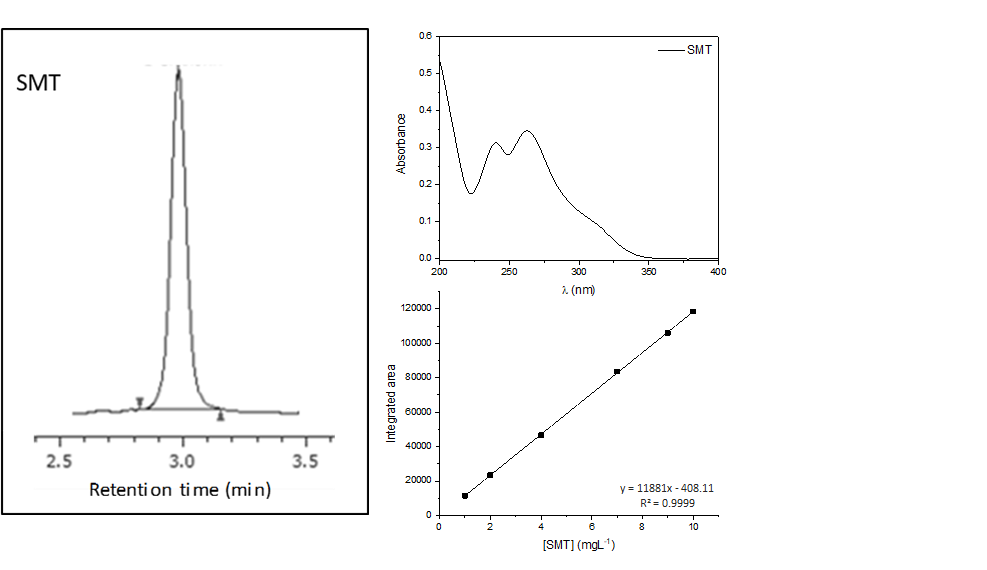


(c)

(b)

(a)

**Fig. S3.** (a) HPLC identification (b) UV-Vis spectrum and (c) standard calibration curve for SMT.

***Ellagic acid (EA)***


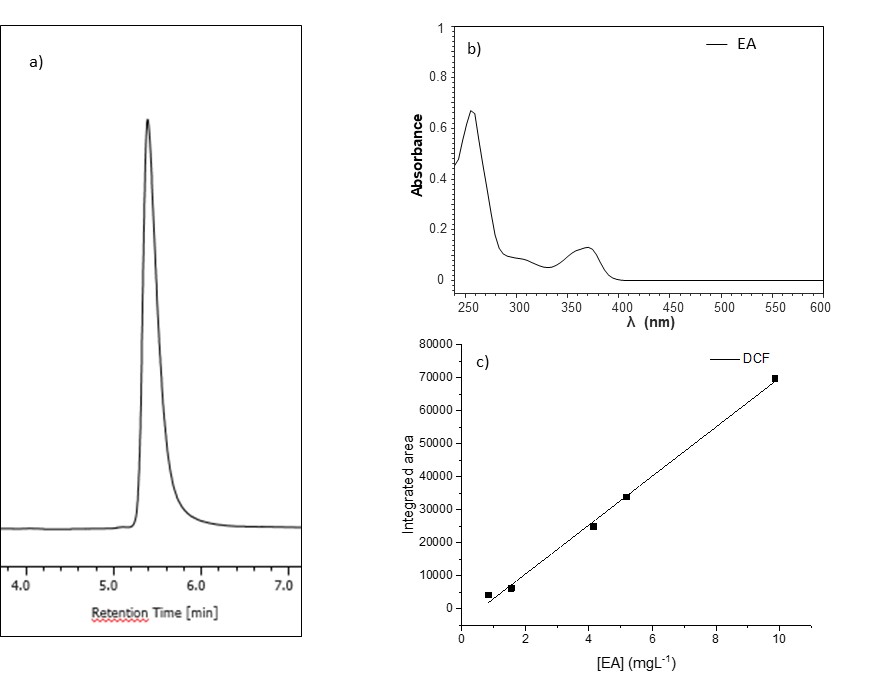


**Fig. S4.** (a) HPLC identification, (b) UV-Vis spectrum and (c) standard calibration curve for EA.

**Table S1** Reported adsorbent materials for the removal of At.

| **Adsorbent** | **Removal (%)** | **Conditions** | | **Refs.** |
| --- | --- | --- | --- | --- |
|  |  | **Initial conc. (mg·L^-1^)** | **Time**  **(h)** |  |
| **SU-101** | 91.5 | 10 | 5 | This work |
| KOH@Ni_8_BDP_6_ | 92-100 | 70 | 5 | [1] |
| M-MWCNTs | 92 | 5 | 1.5 | [2] |
| MPAP | 86.8 | 500 | 0.5 | [3] |
| Ni_8_BDP_6_ | 60 | 1 | 4 | [1] |
| MWCNTs | 58 | 5 | 1.5 | [2] |
| Activated carbon | 57 | 120 | 6 | [4] |
| TMS-SBA-15 | 5 | 0.1 | 2 | [5] |


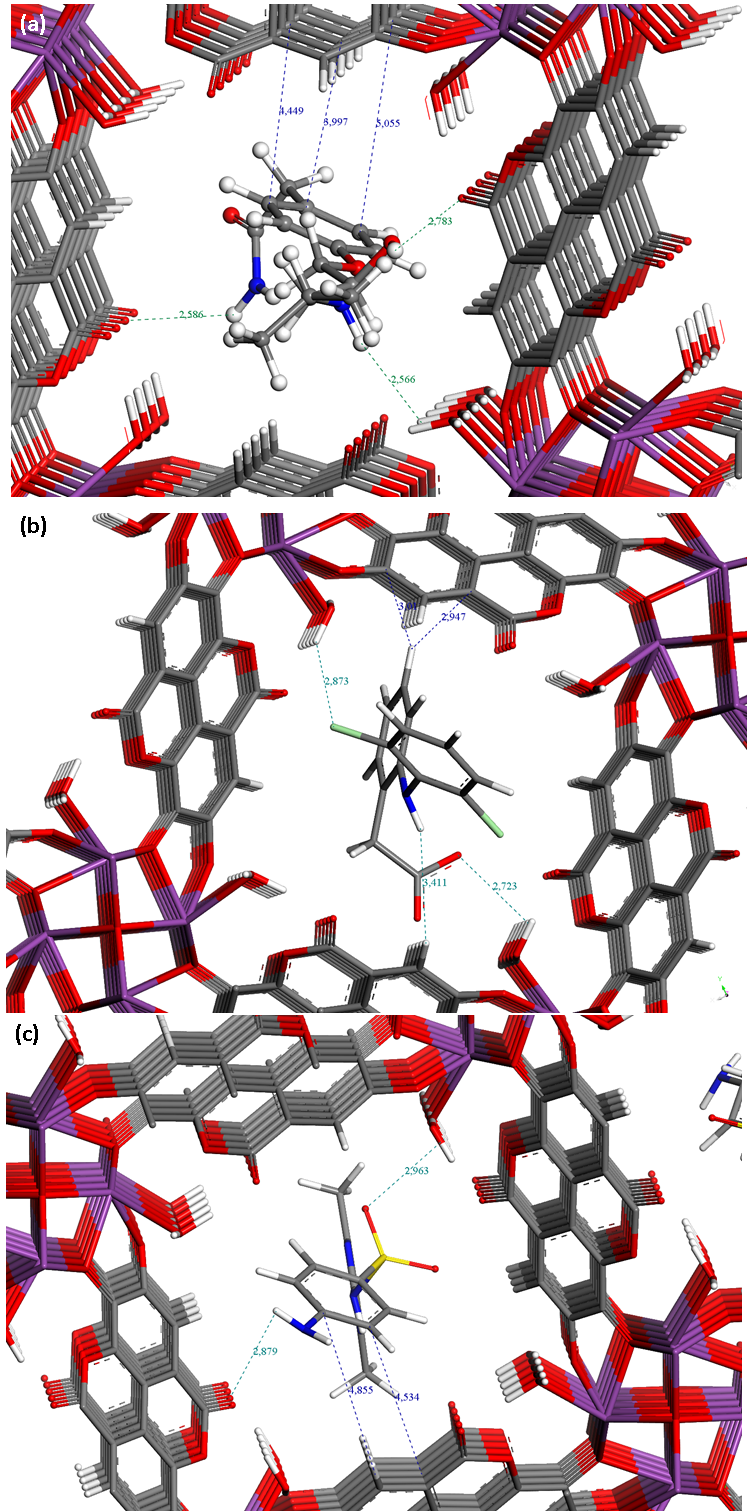


**Fig. S5.** Plausible organization of the contaminant molecules in SU-101 for (a) At, (b) DCF, and (c) SMT. The distances reported in green correspond to hydrogen bonds, while the blue values are the pi-stacking effect.

**Fig. S6.** PXRD patterns of pristine SU-101 (orange) and SU-101 after individual PhAC adsorption experiments: At (red), SMT (blue) and DCF (grey)

**Fig. S7.** FT-IR spectra of SU-101 after individual PhAC adsorption experiments in comparison with the spectra of free DCF, SMT and At compounds.

**Fig. S8.** (a) At adsorbed and desorbed amount on SU-101 and (b) PXRD patterns of SU-101 before and after NaCl treatment.

**Fig. S9.** Photodegradation of the selected PhACs in absence of SU-101 under visible light irradiation.

**Fig. S10.** Kinetic linear fitting plots of the photodegradation studies using individual (solid dark line) and mix (dashed light line) solutions of PhACs. DCF and SMT are represented in black and blue, respectively.

**Table S2.** Photodegradation of DCF and SMT using other reported photocatalysts, including the used experimental conditions.

| **Photocatalyst** | **Drug** | **Removal (%)** | **Conditions**  (Initial conc, time, light source) | **Refs.** |
| --- | --- | --- | --- | --- |
| **SU-101** | **DCF** | 99.8 ± 0.3 | 10 mg L^-1^, 5 h, Vis. | This work |
| PCN-134 |  | >99 | 0.1 mM, 5 h, Vis. | [6] |
| NH_2_-MIL-125(Ti) |  | 100 | 1mg L^-1^, 3 h, UV-Vis. | [7] |
| TiO_2_/g-C_3_N_4_ |  | 93.49 | 5 mg L^-1^, 1.5 h, Vis. | [8] |
| Fe_3_O_4_@MIL-100(Fe) |  | 50.3 | 60 mg L^-1^, 3 h, Vis. | [9] |
| HApTi |  | ̴10 | 8 mg L^-1^, UV-Vis | [10] |
| **SU-101** | **SMT** | 92.5 ± 0.5 | 10 mg L^-1^, 5 h, Vis. | This work |
| MIL-100 (Ti) |  | 100 | 5 mg L^-1^, 4 h, UV-Vis | [11] |
| UIO-66 |  | 77 | 10 mg L^-1^, 24 h, UV-Vis | [11] |
| MIL-125-NH_2_ |  | 70 | 5 mgL^-1^, 5 h, UV-Vis | [11] |
| DCN |  | 39 | 0.1 mM, 1 h, Vis. | [12] |
| MUV-10(Ca) |  | 30 | 5 mg L^-1^, 5 h, UV-Vis | [11] |
| TiO_2_ |  | 30 | 20 mgL-1, 5 h, Vis. | [13] |
| g-C_3_N_4_ |  | 20 | 10 mg L^-1^, 1 h, Vis. | [12] |
| MIL-53(Fe) |  | 12 | 0.02 mM, 1 h, Vis. | [14] |
| IEF-11 |  | 0 | 5 mg L^-1^, 5 h, UV-Vis | [11] |

**Fig. S11.** Band-gap estimation of SU-101 considering an indirect allowed transition.

**Fig. S12.** Comparison between adsorption (dash line) and photodegradation (solid line) experiments of At by SU-101

***LC-MS spectra***

***Tap water.***

**Fig. S13.** Tap water MS used during the photodegradation experiments.

***LC-MS data from At photodegradation at different time intervals***

**Fig. S14.** MS of At photodegradation experiments at different time intervals of 0, 0.25, 0.5, 1, 2, 4, 5 and 24 h.

***LC-MS data from At photodegradation at different time intervals***

**Fig. S14.** MS of At photodegradation experiments at different time intervals of 0, 0.25, 0.5, 1, 2, 4, 5 and 24 h.

***LC-MS data from At photodegradation at different time intervals***

**Fig. S14.** MS of At photodegradation experiments at different time intervals of 0, 0.25, 0.5, 1, 2, 4, 5 and 24 h.

***LC-MS data from At photodegradation at different time intervals***

**Fig. S14.** MS of At photodegradation experiments at different time intervals of 0, 0.25, 0.5, 1, 2, 4, 5 and 24 h.

**Table S3.** Estimated toxicity of At degradation products using the Toxicity Estimation Software Tool (T.E.S.T) based on QSAR model.

|  |  | **At** | **A.1** |
| --- | --- | --- | --- |
| **Oral rat LD_50_** | *Pred. Value (mg/Kg)* | 2382.85 | 2518.30 |
|  | *Pred. Value -Log10 (mol/Kg)* | 2.05 | 2.05 |
| **Bioconcentration factor** | *Pred. Value* | 2.21 | 1.41 |
|  | *Pred. Value -Log10* | 0.34 | 0.15 |
| **Developmental toxicant** | *Pred Value* | Toxicant | Toxicant |
|  | *Pred. Result* | 0.51 | 0.65 |
| **Mutagenicity** | *Pred. Value* | Negative | Negative |
|  | *Pred. Result* | 0.01 | 0.04 |

**Fig. S15.** (a) Comparison of acute toxicity, (b) bioconcentration factors, (c) developmental toxicity and (d) mutagenicity of the generated compounds after At photodegradation.

***LC-MS data from DCF photodegradation at different time intervals***

**Fig. S16.** MS of DCF photodegradation experiments at different time intervals of 0, 0.25, 0.5, 1, 2, 4, 5 and 24 h.

***LC-MS data from DCF photodegradation at different time intervals***

**Fig. S16.** MS of DCF photodegradation experiments at different time intervals of 0, 0.25, 0.5, 1, 2, 4, 5 and 24 h.

***LC-MS data from DCF photodegradation at different time intervals***

**Fig. S16.** MS of DCF photodegradation experiments at different time intervals of 0, 0.25, 0.5, 1, 2, 4, 5 and 24 h.

***LC-MS data from DCF photodegradation at different time intervals***

**Fig. S16.** MS of DCF photodegradation experiments at different time intervals of 0, 0.25, 0.5, 1, 2, 4, 5 and 24 h.

**Table S4.** Estimated toxicity of DCF degradation products using the Toxicity Estimation Software Tool (T.E.S.T) based on QSAR model.

|  |  | **DCF** | **A.2** | **A.3** |
| --- | --- | --- | --- | --- |
| **Oral rat LD_50_** | *Pred. Value (mg/Kg)* | 244 | 355 | 532 |
|  | *Pred. Value -Log10 (mol/Kg)* | 3.08 | 1.59 | 2.39 |
| **Bioconcentration factor** | *Pred. Value* | 26.94 | 0.31 | 0.29 |
|  | *Pred. Value -Log10* | 1.43 | -0.51 | -0.54 |
| **Developmental toxicant** | *Pred Value* | 0.91 | 0.53 | 0.39 |
|  | *Pred. Result* | Toxicant | Toxicant | Non toxicant |
| **Mutagenicity** | *Pred. Value* | 0.53 | 0.08 | 0.21 |
|  | *Pred. Result* | Positive | Negative | Negative |

**Fig. S17.** (a) Comparison of acute toxicity, (b) bioconcentration factors, (c) developmental toxicity and (d) mutagenicity of the generated compounds after DCF photodegradation.

***LC-MS data from SMT photodegradation at different time intervals***

**Fig. S18.** MS of SMT photodegradation experiments at different time intervals of 0, 0.25, 0.5, 1, 2, 4, 5 and 24 h.

***LC-MS data from SMT photodegradation at different time intervals***

**Fig. S18.** MS of SMT photodegradation experiments at different time intervals of 0, 0.25, 0.5, 1, 2, 4, 5 and 24 h.

***LC-MS data from SMT photodegradation at different time intervals***

**Fig. S18.** MS of SMT photodegradation experiments at different time intervals of 0, 0.25, 0.5, 1, 2, 4, 5 and 24 h.

***LC-MS data from SMT photodegradation at different time intervals***

**Fig. S18.** MS of SMT photodegradation experiments at different time intervals of 0, 0.25, 0.5, 1, 2, 4, 5 and 24 h.

**Table S3**. Estimated toxicity of SMT degradation products using the Toxicity Estimation Software Tool (T.E.S.T) based on QSAR model.

|  |  | **SMT** | **A.5** | **A.4** | **A.3** |
| --- | --- | --- | --- | --- | --- |
| **Oral rat LD_50_** | *Pred. Value (mg/Kg)* | 7161 | 3468 | 3199 | 434 |
|  | *Pred. Value -Log10 (mol/Kg)* | 1.59 | 1.24 | 1.57 | 2.34 |
| **Bioconcentration factor** | *Pred. Value* | 2.57 | 0.60 | 0.30 | 7.61 |
|  | *Pred. Value -Log10* | 0.41 | -0.22 | -0.52 | 0.88 |
| **Developmental toxicant** | *Pred Value* | Toxicant | Toxicant | Toxicant | Toxicant |
|  | *Pred. Result* | 0.90 | 0.71 | 0.77 | 0.58 |
| **Mutagenicity** | *Pred. Value* | 0.01 | -0.06 | 0.10 | 0.25 |
|  | *Pred. Result* | Negative | Negative | Negative | Negative |

**Fig. S19.** (a) Comparison of acute toxicity, (b) bioconcentration factors, (c) developmental toxicity and (d) mutagenicity of the generated compounds after SMT photodegradation.

**References:**

[1] S. Rojas, J.A.R. Navarro, P. Horcajada, Metal–organic frameworks for the removal of the emerging contaminant atenolol under real conditions, Dalton Trans. 50 (2021) 2493–2500. https://doi.org/10.1039/D0DT03637D.

[2] B. Dehdashti, M.M. Amin, A. Gholizadeh, M. Miri, L. Rafati, Atenolol adsorption onto multi-walled carbon nanotubes modified by NaOCl and ultrasonic treatment; kinetic, isotherm, thermodynamic, and artificial neural network modeling, J. Environ. Health Sci. Eng. 17 (2019) 281–293. https://doi.org/10.1007/s40201-019-00347-0.

[3] Y. Tang, Z. Chen, Q. Wen, B. Liu, X. Huang, Magnetic powdery acrylic polymer with ultrahigh adsorption capacity for atenolol removal: Preparation, characterization, and microscopic adsorption mechanism, Chem. Eng. J. 446 (2022) 137175. https://doi.org/10.1016/j.cej.2022.137175.

[4] S.C.R. Marques, A.S. Mestre, M. Machuqueiro, A.Ž. Gotvajn, M. Marinšek, A.P. Carvalho, Apple tree branches derived activated carbons for the removal of β-blocker atenolol, Chem. Eng. J. 345 (2018) 669–678. https://doi.org/10.1016/j.cej.2018.01.076.

[5] T.X. Bui, V.H. Pham, S.T. Le, H. Choi, Adsorption of pharmaceuticals onto trimethylsilylated mesoporous SBA-15, J. Hazard. Mater. 254–255 (2013) 345–353. https://doi.org/10.1016/j.jhazmat.2013.04.003.

[6] Y. Gao, J. Xia, D. Liu, R. Kang, G. Yu, S. Deng, Synthesis of mixed-linker Zr-MOFs for emerging contaminant adsorption and photodegradation under visible light, Chem. Eng. J. 378 (2019) 122118. https://doi.org/10.1016/j.cej.2019.122118.

[7] R.R. Solís, A. Gómez-Avilés, C. Belver, J.J. Rodriguez, J. Bedia, Microwave-assisted synthesis of NH_2_-MIL-125(Ti) for the solar photocatalytic degradation of aqueous emerging pollutants in batch and continuous tests, J. Environ. Chem. Eng. 9 (2021) 106230. https://doi.org/10.1016/j.jece.2021.106230.

[8] P. John, K. Johari, N. Gnanasundaram, A. Appusamy, M. Thanabalan, Enhanced photocatalytic performance of visible light driven TiO_2_/g-C_3_N_4_ for degradation of diclofenac in aqueous solution, Environ. Technol. Innov. 22 (2021) 101412. https://doi.org/10.1016/j.eti.2021.101412.

[9] S. Li, J. Cui, X. Wu, X. Zhang, Q. Hu, X. Hou, Rapid in situ microwave synthesis of Fe_3_O_4_@MIL-100(Fe) for aqueous diclofenac sodium removal through integrated adsorption and photodegradation, J. Hazard. Mater. 373 (2019) 408–416. https://doi.org/10.1016/j.jhazmat.2019.03.102.

[10] E. Márquez Brazón, C. Piccirillo, I.S. Moreira, P.M.L. Castro, Photodegradation of pharmaceutical persistent pollutants using hydroxyapatite-based materials, J. Environ. Manage. 182 (2016) 486–495. https://doi.org/10.1016/j.jenvman.2016.08.005.

[11] S. Rojas, J. García-González, P. Salcedo-Abraira, I. Rincón, J. Castells-Gil, N.M. Padial, C. Marti-Gastaldo, P. Horcajada, Ti-based robust MOFs in the combined photocatalytic degradation of emerging organic contaminants, Sci. Rep. 12 (2022) 14513. https://doi.org/10.1038/s41598-022-18590-1.

[12] C. Zhou, Z. Zeng, G. Zeng, D. Huang, R. Xiao, M. Cheng, C. Zhang, W. Xiong, C. Lai, Y. Yang, W. Wang, H. Yi, B. Li, Visible-light-driven photocatalytic degradation of sulfamethazine by surface engineering of carbon nitride：Properties, degradation pathway and mechanisms, J. Hazard. Mater. 380 (2019) 120815. https://doi.org/10.1016/j.jhazmat.2019.120815.

[13] N. Wang, X. Li, Y. Yang, T. Guo, X. Zhuang, S. Ji, T. Zhang, Y. Shang, Z. Zhou, Enhanced photocatalytic degradation of sulfamethazine by Bi-doped TiO2 nano-composites supported by powdered activated carbon under visible light irradiation, Sep. Purif. Technol. 211 (2019) 673–683. https://doi.org/10.1016/j.seppur.2018.10.040.

[14] R. Li, Z. Chen, M. Cai, J. Huang, P. Chen, G. Liu, W. Lv, Improvement of Sulfamethazine photodegradation by Fe(III) assisted MIL-53(Fe)/percarbonate system, Appl. Surf. Sci. 457 (2018) 726–734. https://doi.org/10.1016/j.apsusc.2018.06.294.
